# Supplementary material for: Characterization of genes encoding small heat shock proteins from Bemisia tabaci and expression under thermal stress
Source: PeerJ. 2019 Jun 5;7:e6992. doi: 10.7717/peerj.6992 (PMC6556103; doi:10.7717/peerj.6992)
Supplement: Supplemental Information 1 — The three sHSP genes include Bthsp19.5 (A); Bthsp19.2 (B); and Bthsp21.3 (C). HSP20 family profiles and α-crystallin domains are underscored. [file peerj-07-6992-s001.pdf]

FIGURE S1. Nucleotide and deduced amino acid sequences of genes encoding small heat shock proteins (sHSPs) in *Bemisia tabaci*. The three sHSP genes include *Bthsp19.5* (A); *Bthsp19.2* (B); and *Bthsp21.3* (C). HSP20 family profiles and α-crystallin domains are underscored.

```
1      ATCAACGCAGAGTACATGGGATTCAATAGAAATGGCTTTGCTACCGTACTTGTTGGAAGA
1                                      M  A  L  L  P  Y  L  L  E  E
61     GCTGAACCGACCAACCATCTACGATCAGAACTTTGGTCTAGGATTGTTCAATGATGATTT
11      L  N  R  P  T  I  Y  D  Q  N  F  G  L  G  L  F  N  D  D  F
121    CCCGTCTGCTGTTGGATCTCTGCGCCCTTACTACCTGAGACCATGGAGACTGTTTCCCCA
31      P  S  A  V  G  S  L  R  P  Y  Y  L  R  P  W  R  L  F  P  H
181    CGATGAAAGTGGCATTTCTTCAGTACAGCATGACAAAGATGGATTTAAGGTGAATTTAGA
51      D  E  S  G  I  S  S  V  Q  H  D  K  D  G  F  K  V  N  L  D
241    CGTACAGCAATTCAAGCCAGAGGAGGTCAACGTGAAAATAGCTGACAACTATGTCATCGT
71      V  Q  Q  F  K  P  E  E  V  N  V  K  I  A  D  N  Y  V  I  V
301    CAACGCTAAACATGAGGAGCGTAGTGATGAACATGGCTTCATTTCACGAGAATTCACCCG
91      N  A  K  H  E  E  R  S  D  E  H  G  F  I  S  R  E  F  T  R
361    CCGTTACCTTCTGCCAAAAGACGTAAACGCTGAGGCCTTGACTTCATCTCTCTCTTCCGA
111     R  Y  L  L  P  K  D  V  N  A  E  A  L  T  S  S  L  S  S  D
421    CGGAGTTCTTTCCATCCAGGCTCCACCAAAGGCAATCACCAACGATAAGGGAAATGAGCG
131     G  V  L  S  I  Q  A  P  P  K  A  I  T  N  D  K  G  N  E  R
481    TCAAATTCCAGTCACCAAAACCAACGCCCCAGCAATCAAACAACAACAACAACAACAGAA
151     Q  I  P  V  T  K  T  N  A  P  A  I  K  Q  Q  Q  Q  Q  Q  K
541    GAAGTAAATTATCGTGAATGACATTTACTCTCATCTTAAGTATTTATTTTTACGTCATCT
171     K  *
601    TTCTAAGGTGAATATCTCTTACATTTTTAATGTTACTATGTAACCGACTATGTTATTACT
661    CTTTATGATAATCTAATTTTAAGTTCCTATCCATGAATAGTTTTTAATTTAGTCCCATTT
721    CAAATTTTGTCTGACTGTACATGTGTCCATTCATTAATCTTCAAAATATTAAGTTACAAA
781    TTTTCATCTTTCATACTTATTAAGGAGAAAATGTTTTCAACCGTATGCAGAGCTAGACCA
841    TTCATTGTCACATTTTACTGAATTTGACATCTTCTCAGGGATATATAGGATTCAAATTTT
901    CATCTAGTTTAGTCTAGCTGTGCTCTCTGAGAAATATGAATATTAAGTGAAATTAAGTAC
961    AATTTTCTTTCTTGTTATTCTTTTTGAACTCTCAACTTGGATCAGTAAAATGTCATTCAA
1021   TACTCAAATAATTACTTAATGCTGTTGAATTATTCTGTTCAACTGTTAAAGTCATCAGAC
1081   TTCACCCTAGTTAGTGTATTAAGATTGTTTGTATAAATTATATTGTAAGGAAGATTTATT
1141   ACTTTTTAATAAAGGTGTCCAAAAAAAAAAAAAAAAAAAAAAAAAAAAAAAA
```

A

```
1      AGCGGTTCGATAAATCCACTGTGTATGTCTCTCTTGCCCTACTTGTTGGAAGAATTGAAC
1                                    M  S  L  L  P  Y  L  L  E  E  L  N
61     CGTCCAACAGTGTATGACCAAAACTTCGGTGTTGGACTGTGGAATGATGATCTGCCATCT
13      R  P  T  V  Y  D  Q  N  F  G  V  G  L  W  N  D  D  L  P  S
121    ATCACTGCCATTCGCCCGTTTTATATGCGGCCATGGAGACTGCTTCCCCAAAATGAGAGT
33      I  T  A  I  R  P  F  Y  M  R  P  W  R  L  L  P  Q  N  E  S
181    GGGACTTCATCAGTACAGCACGATAAAGATGGATTTAAGGTGAACTTAGATGTGCAGCAG
53      G  T  S  S  V  Q  H  D  K  D  G  F  K  V  N  L  D  V  Q  Q
241    TTCAAACCTGAAGAAGTGAATGTCAAAGTCAGTGACAATTATGTTACCATCCACGCCAAA
73      F  K  P  E  E  V  N  V  K  V  S  D  N  Y  V  T  I  H  A  K
301    CACGAAGAACGCAGTGATGAGCATGGTTTTATTTCTCGGGAATTCACTAGGCGTTATCTC
93      H  E  E  R  S  D  E  H  G  F  I  S  R  E  F  T  R  R  Y  L
361    ATCCCTAAAGACGTCAATGCGGAAGCCTTGACTTCAAGTCTTTCATCTGATGGTGTTCTC
113     I  P  K  D  V  N  A  E  A  L  T  S  S  L  S  S  D  G  V  L
421    TCTGTACATGCTCCGCCAAAGGCTATCACCAATGAGAATGGCAGTGAGCGCCAAATTCCA
133     S  V  H  A  P  P  K  A  I  T  N  E  N  G  S  E  R  Q  I  P
481    ATTACTAAAACCAATGCTCCCGCAATCAAACAGGAACAAGCGAAGAAGAAGTAATCAATC
153     I  T  K  T  N  A  P  A  I  K  Q  E  Q  A  K  K  K  *
541    CTACCTTGATGTCTAATTGAGTTTTCCTCTTTCTCCTCAATGAGCTTCTTCCTTTTTCCT
601    TGTACTGTAAATATTTTCACTTACTTATTTTTGTTCCTATCATTGAGAACCAGCTTGTAT
661    CAAGAAATGTTAAACAGTGAAAGTGAAACATAAATTCCCTTTTAAATACTATTATGCCCC
721    GTCAATAATTGTACTATCGTTTTCACGGAGCAATAGTAAAATTCCTAAAAAAACCATGTA
781    TTTTTCATAACAGGATATCATCGTAAGTGTTGTGTAAGCAGAGTGCTACAGTTAGCACGT
841    GTTCTCTTTTCACCACCACCAAGAAATTACCACCGTGTGTTGTACTTATTTAACTTTTAG
901    AACTATTTTTAAGTCTGCTCCATTGTTACATTATACAAAGTAGTGATAAGAAATTCACTT
961    GGAAGGTATTTTGTTCTAGGTTATTGTCAACATGAAATGAATTACTGTACTGTTCTTGTG
1021   AGACTGCTACTATTTATGTAAATTTTATTATTTTTTTAAATAAAATTTCCAGACAAAAAA
1081   AAAAAAAAAAAAAAAAAAAAAAAAAAA
```

B

1	AGCGGTTCGATAAATCCACTGTGATATCTTCTGAGTTCGATACGTAGGTTGCGCAACCCT
61	CGAGAGGGTATACCAGCTGGACGTCCGCCGGCGCTTAAGTGTATGTCTCTCTTGCCCTAC
1	M S L L P Y
121	TTGTTGGAAGAATTGAACCGTCCAACAGTGTATGACCAAAACTTCGGTGTTGGACTGTGG
7	L L E E L N R P T V Y D Q N F G V G L W
181	AATGATGATCTGCCATCTATCACTGCCATTCGCCCGTTTTATATGCGGCCATGGAGACTG
27	N D D L P S I T A I R P F Y M R P W R L
241	CTTCCCCAAAATGAGAGTGGGACTTCATCAGTACAGCACGATAAAGATGGATTTAAGGTG
47	L P Q N E S G T S S V Q H D K D G F K V
301	AACTTAGATGTGCAGCAGTTCAAACCTGAAGAAGTGAATGTCAAAGTCAGTGACAATTAT
67	N L D V Q Q F K P E E V N V K V S D N Y
361	GTTACCATCCACGCCAAACACGAAGAACGCAGTGATGAGCATGGTTTTATTTCTCGGGAA
87	V T I H A K H E E R S D E H G F I S R E
421	TTCACTAGGCGTTATCTCATCCCTAAAGACGTCAATGCGGAAGCCTTGACTTCAAGTCTT
107	F T R R Y L I P K D V N A E A L T S S L
481	TCATCTGATGGTGTTCTCTCTGTACATGCTCCGCCAAAGGCTATCACCAATGAGAATGGC
127	S S D G V L S V H A P P K A I T N E N G
541	AGTGAGCGCCAAATTCCAATTACTAAAACCAATGCTCCCGCAATCAAACAGGAACAAGCG
147	S E R Q I P I T K T N A P A I K Q E Q A
601	AAGAGAGCAGAAACGCCAACACCAACGATGCCGTCACCGCTAACAACAAGCCCGACAGCG
167	K R A E T P T P T M P S P L T T S P T A
661	AAGAAAGATTGAACGAATTCGTCCGCAAATCCTACGCCGGAATCAACAGTGCCTTCGTCA
187	K K D *
721	ACGCCTTCGACTTCTCTGCCCAATTCCTTGGACAAAAGGAAGCCGACTACGTTTGCACCT
781	TTGCCTTCGCCCGCAGCCCAGTTGCTGAGAAATCTCGTTTCCTCTTCTACGGACACTACA
841	ACACTGCCAACAACAAGAAACAACAGTGCGCTTTCCACGCCTCCGCTGAGATGCCCAATG
901	TTCCCTTGACCAACCCTGCTGCCGCCATGAAGGCTGAGCCAGCTTCCAAGATCTACGCTA
961	ACTTCAAGTTCGGAGAATCTTTCGAAAATGCCGCCAAGGTTCACTTCAACGCTAACTTGA
1021	AGCAGAGCTCTGAACGCCGTCAATTCCTCCGCAACAACGCCCTCTACAAGCAATGCGAAT
1081	CCGAAATGGAACGTGGACAATACTTCCTCCCTGCTTGCCGTAACTTCACCGTTGCTGACA
1141	ACAGAATGAACGAATACTACTACAACTTCAACTTCCAAAAAAAAAAAAAAAAAAAAAAAA
1201	AAAAAAAAA

C
